# Supplementary material for: Perception of COVID-19 Booster Dose Vaccine among Healthcare Workers in India and Saudi Arabia
Source: Int J Environ Res Public Health. 2022 Jul 22;19(15):8942. doi: 10.3390/ijerph19158942 (PMC9332579; doi:10.3390/ijerph19158942)
Supplement: Supplementary file 1 [file ijerph-19-08942-s001.zip › ijerph-1784105-supplementary.pdf]

PERCEPTION OF COVID-19 BOOSTER DOSE VACCINE – QUESTIONNAIRE

Age	☐ 18-30yrs	☐ 31-40yrs	☐ >40 yrs.
Sex	☐ Male	☐ Female	
Education	☐ Undergraduate	☐ Postgraduate	☐ PhD
Residence	☐ Rural	☐ Urban	
Have you received all the necessary vaccines in your lifetime?	☐ Yes	☐ No	
Do you know about the COVID-19 vaccine?	☐ Yes	☐ No	☐ Don't Know
Do you believe that the COVID-19 vaccine is safe?	☐ Yes	☐ No	☐ Don't Know
Have you received two doses of COVID-19 vaccines?	☐ Yes	☐ No	☐ Don't Know
Are you willing to take the COVID-19 booster vaccine without any hesitation?	☐ Yes	☐ No	☐ Don't Know
Reasons for not willing to take COVID-19 vaccine (if the previous answer is NO)	☐ I am concerned as I don't know enough about the vaccine		
	☐ I am concerned about the short-term side effects (e.g., fever, etc.)		
	☐ I am concerned about possible long-term side effects		
	☐ I am concerned because I don't think the vaccine will be effective		
	☐ I am against vaccines in general		
Do you think that COVID-19 booster vaccination has adverse reactions?	☐ Yes	☐ No	☐ Don't Know
Do you encourage your family/friends/relatives to get the booster COVID-19 vaccine?	☐ Yes	☐ No	☐ Don't Know
Do you believe the COVID-19 booster vaccine can reduce the spread of COVID-19?	☐ Yes	☐ No	☐ Don't Know
Do you believe the COVID-19 booster vaccine can reduce the complications associated with COVID- 19?	☐ Yes	☐ No	☐ Don't Know
Do you think that if everyone in society maintains the preventive measures, the COVID-19 pandemic can be eradicated without vaccination?	☐ Yes	☐ No	☐ Don't Know
Do you think Pharmaceutical companies have developed safe and effective COVID-19 vaccines?	☐ Yes	☐ No	☐ Don't Know
Have you received the COVID-19 booster dose because it is mandatory?	☐ Yes	☐ No	☐ Don't Know
Do you think Mix-Matching the booster dose is safe and effective?	☐ Yes	☐ No	☐ Don't Know
Do you believe only high-risk individuals such as health care workers and elderly persons with other diseases need booster dose?	☐ Yes	☐ No	☐ Don't Know
